# Genetic regulators of sputum mucin concentration and their associations with COPD phenotypes

**DOI:** 10.1371/journal.pgen.1010445

**Published:** 2023-06-23

**Authors:** Eric Van Buren, Giorgia Radicioni, Sarah Lester, Wanda K. O’Neal, Hong Dang, Silva Kasela, Suresh Garudadri, Jeffrey L. Curtis, MeiLan K. Han, Jerry A. Krishnan, Emily S. Wan, Edwin K. Silverman, Annette Hastie, Victor E. Ortega, Tuuli Lappalainen, Martijn C. Nawijn, Maarten van den Berge, Stephanie A. Christenson, Yun Li, Michael H. Cho, Mehmet Kesimer, Samir N. P. Kelada

**Affiliations:** 1 Department of Biostatistics, University of North Carolina, Chapel Hill, North Carolina, United States of America; 2 Department of Biostatistics, Harvard T.H. Chan School of Public Health, Boston, Massachusetts, United States of America; 3 Marsico Lung Institute, University of North Carolina, Chapel Hill, North Carolina, United States of America; 4 Department of Genetics, University of North Carolina, Chapel Hill, North Carolina, United States of America; 5 New York Genome Center, New York, New York, United States of America; 6 Department of Systems Biology, Columbia University, New York, New York, United States of America; 7 Division of Pulmonary, Critical Care, Allergy, & Sleep Medicine, Department of Medicine, University of California San Francisco, San Francisco, California, United States of America; 8 Pulmonary & Critical Care Medicine Division, University of Michigan, Ann Arbor, Michigan, United States of America; 9 Medical Service, VA Ann Arbor Healthcare System, Ann Arbor, Michigan, United States of America; 10 Breathe Chicago Center, University of Illinois, Chicago, Illinois, United States of America; 11 Channing Division of Network Medicine, Brigham and Women’s Hospital and Harvard Medical School, Boston, Massachusetts, United States of America; 12 VA Boston Healthcare System, Jamaica Plain, Massachusetts, United States of America; 13 Department of Internal Medicine, Wake Forest School of Medicine, Winston-Salem, North Carolina, United States of America; 14 Department of Internal Medicine, Division of Respiratory Medicine, Mayo Clinic, Scottsdale, Arizona, United States of America; 15 Department of Pathology and Medical Biology, University of Groningen, University Medical Center Groningen, Groningen, the Netherlands; 16 Groningen Research Institute for Asthma and COPD, University Medical Center Groningen, Groningen, the Netherlands; 17 Department of Pulmonary Diseases, University of Groningen, University Medical Center Groningen, Groningen, the Netherlands; HudsonAlpha Institute for Biotechnology, UNITED STATES

## Abstract

Hyper-secretion and/or hyper-concentration of mucus is a defining feature of multiple obstructive lung diseases, including chronic obstructive pulmonary disease (COPD). Mucus itself is composed of a mixture of water, ions, salt and proteins, of which the gel-forming mucins, MUC5AC and MUC5B, are the most abundant. Recent studies have linked the concentrations of these proteins in sputum to COPD phenotypes, including chronic bronchitis (CB) and acute exacerbations (AE). We sought to determine whether common genetic variants influence sputum mucin concentrations and whether these variants are also associated with COPD phenotypes, specifically CB and AE. We performed a GWAS to identify quantitative trait loci for sputum mucin protein concentration (pQTL) in the Sub-Populations and InteRmediate Outcome Measures in COPD Study (SPIROMICS, n = 708 for total mucin, n = 215 for MUC5AC, MUC5B). Subsequently, we tested for associations of mucin pQTL with CB and AE using regression modeling (n = 822–1300). Replication analysis was conducted using data from COPDGene (n = 5740) and by examining results from the UK Biobank. We identified one genome-wide significant pQTL for MUC5AC (rs75401036) and two for MUC5B (rs140324259, rs10001928). The strongest association for MUC5B, with rs140324259 on chromosome 11, explained 14% of variation in sputum MUC5B. Despite being associated with lower MUC5B, the C allele of rs140324259 conferred increased risk of CB (odds ratio (OR) = 1.42; 95% confidence interval (CI): 1.10–1.80) as well as AE ascertained over three years of follow up (OR = 1.41; 95% CI: 1.02–1.94). Associations between rs140324259 and CB or AE did not replicate in COPDGene. However, in the UK Biobank, rs140324259 was associated with phenotypes that define CB, namely chronic mucus production and cough, again with the C allele conferring increased risk. We conclude that sputum MUC5AC and MUC5B concentrations are associated with common genetic variants, and the top locus for MUC5B may influence COPD phenotypes, in particular CB.

## Author summary

Chronic obstructive pulmonary disease (COPD) is characterized by presence of emphysema and/or chronic bronchitis. Excessive mucus production is a defining phenotype of chronic bronchitis, and is associated with several important features of COPD, including exacerbations and loss of lung function. Recent studies have demonstrated that the amount of mucus produced in COPD patients is an important marker of disease state. We investigated whether common genetic variants are associated with the concentration of two key proteins in mucus, MUC5AC and MUC5B, and whether the variants we identified are also associated with COPD outcomes. We identified multiple genetic variants that were associated with MUC5AC or MUC5B concentration. The strongest association we detected, for MUC5B on chromosome 11, was also associated with features of COPD, including chronic bronchitis and acute exacerbations, in one COPD study population but not another. Results from a much larger study, the UK Biobank, indicate that this variant is associated with chronic mucus production and chronic cough, which are key features of chronic bronchitis. Thus, we conclude that the concentration of key proteins in mucus are influenced by genetic variation, and that a variant on chromosome 11 that affects MUC5B may in turn alter COPD outcomes.

## Introduction

Chronic obstructive pulmonary disease (COPD) is a smoking-related disease that affects more than 200 million people and is the fourth leading cause of death worldwide [1,2]. The disease is characterized by the presence of emphysema and/or chronic bronchitis (CB). Chronic mucus hyper-secretion is a defining phenotype of CB and is associated with airway obstruction due to mucus plugs [3], acute exacerbations (AE) [4], and accelerated loss of lung function over time [5,6].

Mucus itself is composed of a mixture of water, ions, salt and proteins, and mucin glycoproteins, most prominently the gel-forming mucins, MUC5AC and MUC5B. Their concentrations and biochemical properties (e.g., size and oxidation state) largely determine the viscoelastic properties of mucus in health and disease [7]. Recent studies emanating from the Sub-Populations and InteRmediate Outcome Measures in COPD Study (SPIROMICS) have provided compelling evidence that the concentration of mucin proteins in induced sputum is an important biomarker in COPD [8,9]. Kesimer et al. showed that mucin concentrations (total, MUC5AC and/or MUC5B) was associated with smoking history, phlegm production, CB, risk of AE, and disease severity [8]. A subsequent study showed that the concentration of MUC5AC was associated with disease initiation and progression [9].

Here, we report our findings of a genome-wide search for common genetic variants associated with variation in sputum mucin protein concentration, that is, mucin protein quantitative trait loci (pQTL). Previous studies have identified genetic variants associated with mucin gene expression (eQTL) that are located within or near *MUC5AC* [10–12] (in asthma) and *MUC5B* (in idiopathic pulmonary fibrosis, IPF [13]). That said, we conducted a genome-wide search for mucin pQTL because our prior work in a mouse model system indicated distal (or trans) pQTL for mucins were possible and perhaps even likely [14]. We leveraged quantitative mass spectrometry-based measurements of induced sputum samples from SPIROMICS that were generated previously [8,9] to identify main effect pQTL and pQTL that result from genotype × smoking interactions. Subsequently, we tested whether the pQTL we identified were associated with COPD outcomes, namely CB and AE, in SPIROMICS, followed by replication analysis in COPDGene and the UK Biobank.

## Results

### GWAS for Mucin pQTL

We conducted a GWAS of total and specific (MUC5AC and MUC5B) mucin concentrations in sputum to identify novel regulators of these biomarkers in SPIROMICS. Descriptive statistics of study participants are provided in Table 1. The mucin phenotype data represent a subset of subjects described in two previous studies [8,9], and comprise a subset of participants in SPIROMICS (S1 Fig). In this sample, there was a clear effect of smoking history on total mucin concentration (S2 Fig), but among COPD cases, there was not a linear or monotonically increasing relationship between total mucin concentration and disease severity (as reflected by Global Initiative for Chronic Obstructive Lung Disease (GOLD) stage). Similar patterns were observed for MUC5AC and MUC5B (S2 Fig).

**Table 1:** Descriptive Statistics of SPIROMICS Participants in Mucin GWAS.*

	Non-smoking Controls		At-risk		GOLD 1		GOLD 2		GOLD 3	
Study Population	Total Mucin	MUC5AC, MUC5B	Total Mucin	MUC5AC, MUC5B	Total Mucin	MUC5AC, MUC5B	Total Mucin	MUC5AC, MUC5B	Total Mucin	MUC5AC, MUC5B
N	50	25	219	46	130	40	241	56	67	48
Age, mean (range)	57.9 (40–80)	61.3 (42–80)	60.4 (40–79)	63.3 (40–79)	66.7 (45–80)	65.8 (49–79)	65.0 (42–80)	64.9 (48–80)	66.5 (48–80)	67.1 (52–80)
Males, n (%)	26 (51.0)	13 (52.0)	117 (53.2)	21 (45.7)	91 (70.0)	31 (77.5)	142 (58.9)	35 (62.5)	35 (52.2)	21 (43.8)
European Ancestry, n (%)	36 (72.0)	25 (100)	155 (70.8)	46 (100)	112 (86.2)	40 (100)	214 (88.8)	56 (100)	59 (88.1)	48 (100)
African Ancestry, n (%)	14 (28.0)	NA^†^	64 (29.2)	NA^‡^	18 (13.9)	NA^‡^	27 (11.2)	NA^‡^	8 (11.9)	NA^‡^
Chronic Bronchitis^†^, n (%)	3 (6.0)	2 (8.0)	89 (40.6)	15 (32.6)	52 (40.0)	19 (47.5)	134 (55.4)	35 (62.5)	29 (43.2)	20 (41.7)
Current smoker, n (%)	0 (0)	0 (0)	113 (51.6)	21 (45.7)	45 (34.6)	17 (42.5)	114 (47.1)	26 (46.4)	18 (26.9)	11 (22.9)
Former smoker, n (%)	0 (0)	0	106 (48.4)	25 (54.4)	85 (65.4)	23 (57.5)	127 (52.5)	30 (53.6)	49 (73.3)	37 (77.1)
Smoking, pack-years (range)	0	0 (0)	43.0 (20–150)	44.0 (20–100)	51.9 (20–160)	54.7 (20–117)	57.1 (20–270)	55.8 (20–147)	51.8 (20–126)	50.3 (21–126)

*One subject with a GOLD stage of 4 was included in the total mucin analysis but is not shown here.

^†^ Based on St. George’s Respiratory Questionnaire

^‡^Due to limited sample size of SPIROMICS participants of African ancestry with MUC5AC/MUC5B data, only data on European ancestry subjects were used in these analyses.

**Fig 1:**
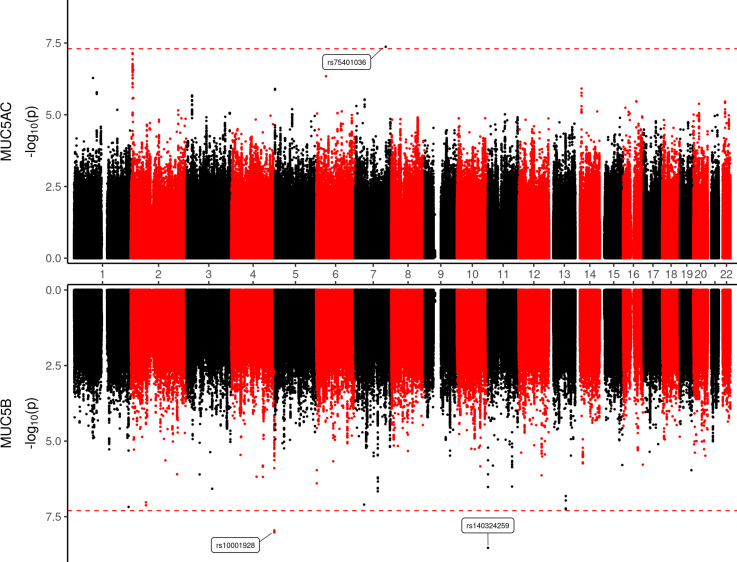
Distal and local pQTL for sputum MUC5AC and MUC5B concentration. Results of association analysis using sputum mucin concentration data from 215 EA SPIROMICS participants are shown. Dashed red line denotes genome-wide significance threshold.

We did not detect any genome-wide significant loci (p<5.0x10^-8^) associated with total mucin concentration based on data from SPIROMICS participants of European ancestry (EA, N = 576) or African ancestry (AA, N = 132) participants (S3 and S4 Figs), nor in combined analysis of EA and AA subjects (S5 Fig). Testing for joint effects of SNP and SNP × smoking (pack-years) interactions did not reveal any loci associated with total mucin concentration either. In contrast, despite relatively small sample size (N = 215 EA subjects), we identified three genome-wide significant pQTL for MUC5AC or MUC5B (Figs 1, S6 and S7 and S1 Table). In addition to the pQTL for MUC5AC on chromosome (Chr) 7 (rs75401036), we identified one highly suggestive locus on Chr 2 (rs16866419, p = 7.2x10^-8^), thus both MUC5AC pQTL are located on chromosomes other than Chr 11 where *MUC5AC* and *MUC5B* are located (i.e., act in trans). We note that restricting our analysis to variants located in/near MUC5AC, i.e., with a reduced multiple testing correction, did not reveal any local pQTL for MUC5AC, and this includes testing variants previously associated with *MUC5AC* gene expression [10–12] including rs12788104, rs11602802, rs11603634, rs1292198170, and rs1132436. Overall, SNP-based heritability estimates for MUC5AC (*h*^2^_SNP_ = 0.712; S.E. = 1.322) and MUC5B (*h*^2^_SNP_ = 0.608, S.E. = 1.475) were high but imprecise, which is not surprising given the relatively small sample size.

**Fig 2:**
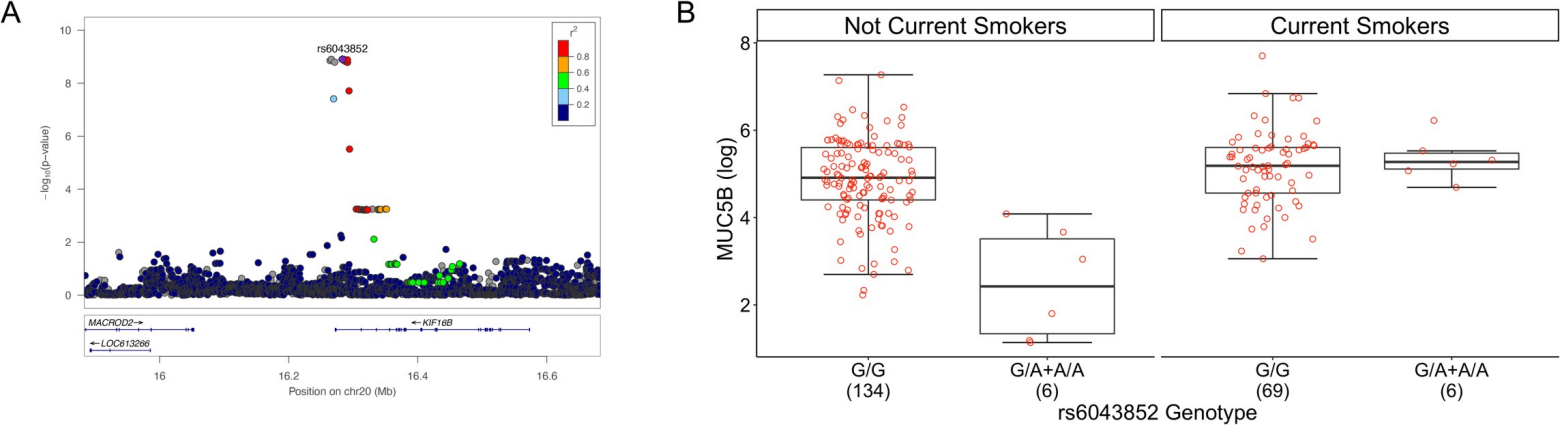
A genotype x smoking interaction locus (rs6043852) for sputum MUC5B concentration on Chromosome 20. **A**. Locus zoom plot for the genotype x smoking locus (rs6043852). **B**. MUC5B concentration as a function of both rs6043852 genotype and current smoking status. The not current smoker category includes never smokers and former smokers. Numbers in parentheses on x-axis denote sample size per genotype. Note that while we plot carriers of the minor allele here as one group, the regression model for MUC5B used genotype dosages.

For MUC5B, one local pQTL was detected on Chr 11 (rs140324259), and one distal pQTL was located on Chr 4 (rs10001928). One additional MUC5B pQTL (rs6043852), located in the intron of *KIF16B* on chromosome 20 was detected by testing for the joint effects of SNP + SNP × smoking interactions (joint test p-value = 1.3x10^-9^, Fig 2A). Further analysis revealed that the interaction itself contributed substantially to the joint effect (p_interaction_ = 1.1x10^-7^), such that rs6043852 was associated with MUC5B concentration only in subjects that are not current smokers (Fig 2B). Given the relatively low minor allele frequency of rs6043852 (3%), the number of subjects harboring genotypes with the minor allele (A) and of contrasting smoking status was not large (n = 6 A allele carriers in current smokers and n = 6 in the combined never plus former smoker group). Hence this SNP × smoking interaction pQTL must be interpreted with caution.

**Fig 3:**
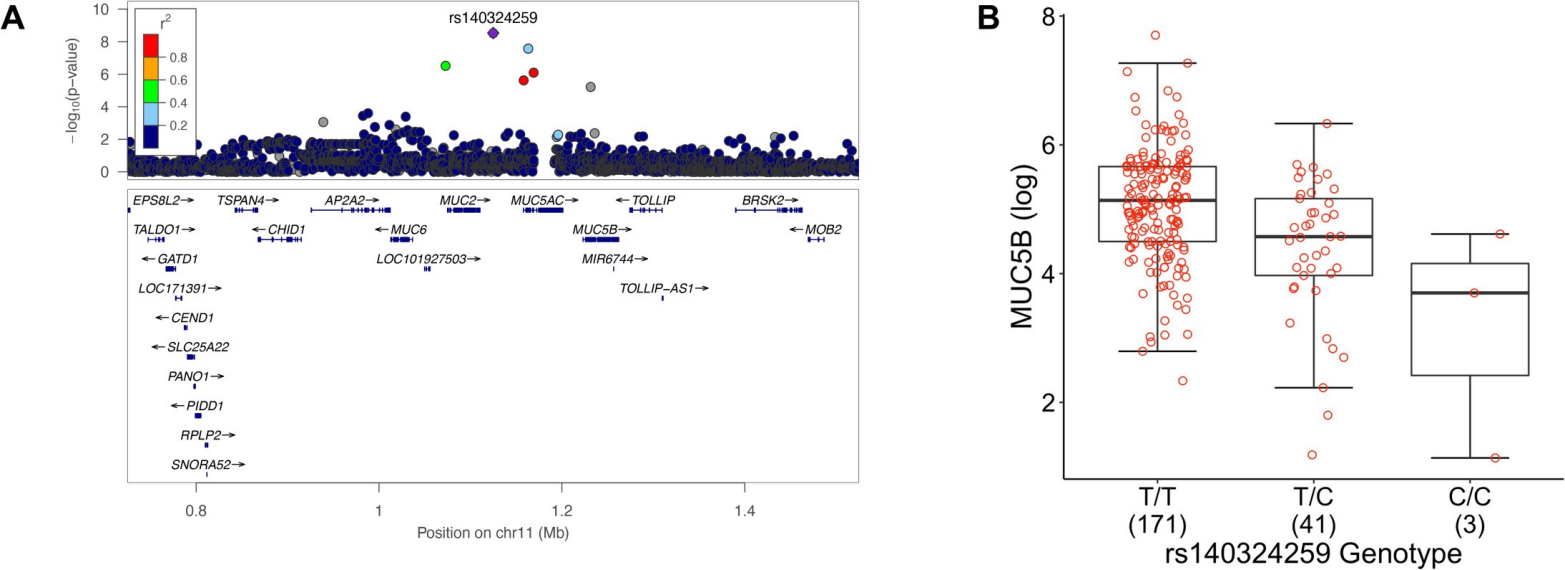
The chromosome 11 MUC5B pQTL. **A**. Regional view of association test results. Four genes not shown due to small size. The lead variant, rs140324259, is approximately 100 kb upstream of *MUC5B*. **B.** Effect of rs140324259 genotype on sputum MUC5B concentration. Numbers in parentheses on x-axis denote sample size per genotype. Each C allele yields a 0.8 log (ln) unit decreased in MUC5B, equating to a 2.3 picomol/ml decrease in MUC5B concentration.

The strongest pQTL we identified was for MUC5B on Chr 11. The lead variant, rs140324259, is located approximately 100 kb upstream of *MUC5B*, in between *MUC2* and *MUC5AC* (Fig 3A). A second variant located in intron 6 of *MUC5AC*, rs28668859, was also associated with MUC5B concentration; conditional analysis revealed this this signal was partially dependent on linkage disequilibrium (LD, R^2^ = 0.20) with rs140324259 (conditional p-value = 1.6x10^-3^). Neither rs140324259 nor rs28668859 are in LD (R^2^ = 0.02 and 0.01, respectively) with the *MUC5B* promoter variant rs35705950 that is a well-known *MUC5B* eQTL and is associated with IPF [13]. After adjusting for covariates, rs140324259 genotype explained ∼14% of variation in sputum MUC5B, and each minor allele (C) contributed a ∼2.3 pmol/ml unit decrease in MUC5B (Fig 3B), an effect size that is greater than the effect of current smoking status (yes vs. no, ∼1.4 pmol/ml). Adjusting for disease severity (using GOLD stage) did not materially change these results.

We asked whether lead MUC5B pQTL, rs140324259, was associated with *MUC5B* gene expression in SPIROMICS or other studies. In a subset of SPIROMICS participants (n = 144) for whom airway brush RNA-seq data exist [15], we used a tagSNP for rs140324259, namely rs55680540 (LD R^2^ = 0.72 in entire SPIROMICS population), but found no correlation between genotype and *MUC5B* expression (S8A Fig). No other variants in the region were significantly associated with *MUC5B* expression (S8B Fig). Additionally, rs140324259 was also not associated with *MUC5B* expression in the nasal epithelium of subjects with cystic fibrosis [16] or asthma [12], nor was it reported as an eQTL in any tissue in the GTEx dataset [17], including homogenized lung tissue.

Given that power for eQTL detection could be an issue underlying the negative eQTL association results, we asked whether rs140324259 or four variants in LD (rs55680540, rs28668859, rs11604917, and rs76498418) could potentially affect gene expression by altering transcription factor binding or chromatin state using Haploreg [18]. As shown in S2 Table, rs140324259, rs11604917, and rs55680540 are predicted to alter binding of transcription factors, and there is some evidence that rs11604917 and rs55680540 alter chromatin state in relevant cell types or tissues (S3 Table). Perhaps most notably, rs11604917 lies in an enhancer region in multiple cell types and tissues and is predicted to alter binding of the transcription factor RBP-J. The alternate allele (C) of rs11604917 disrupts the consensus sequence at the first position of an almost invariant motif (S9 Fig). Given that RBP-J is part of the Notch signaling pathway that determines ciliated vs. secretory cell fate in murine airways [19], we asked whether rs11604917 was associated with the frequencies of basal, secretory and ciliated epithelial cells, which were estimated using a deconvolution approach on airway brush bulk RNA-seq data from 137 SPIROMICS participants (largely overlapping with the eQTL dataset described above). We applied a previously developed cell type deconvolution method [20] that was shown to perform well on airway samples [21] (see Methods). We did not detect any associations between rs11604917 genotype and frequencies of these cell types, though smoking status was strongly associated with cell type proportions (S4 Table).

### Association of MUC5B pQTL with COPD Phenotypes

**Fig 4:**
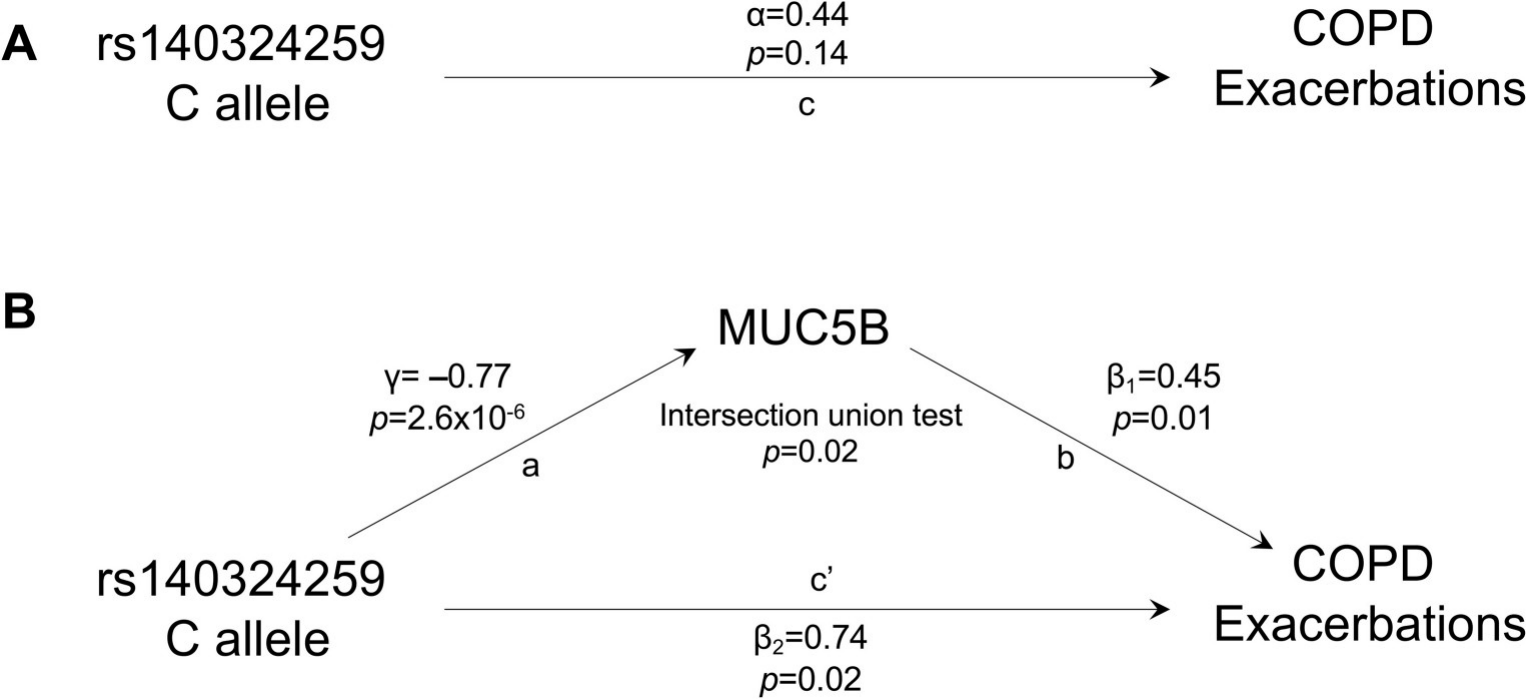
Mediation analysis reveals that rs140324259 exerts effects on exacerbations in the year prior to enrollment through direct and indirect paths with contrasting allele effects. We leveraged the mediation analysis framework of Baron and Kenny [22] to examine whether rs140324259 exerts effects on exacerbations through MUC5B. Using complete data on 142 subjects, in **(A)** we tested for the total effect of rs140324259 on acute exacerbations of COPD (“c”). In (**B)**, the mediation analysis framework is shown in which the effect of rs140324259 on acute exacerbations is modeled as the sum of direct (rs140324259 to exacerbations, c’) and indirect paths (rs140324259 to exacerbations via MUC5B (a, b)). Statistical evidence of the indirect path assessed by jointly testing that both rs140324259 → MUC5B (a) and MUC5B → exacerbations (b) are significant using an intersection union test (which is equivalent to testing that γ x β_1_ is not equal to 0). β_1_ (b) and β_2_ (c’) come from the same negative binomial regression model including both rs140324259 and MUC5B as predictors of exacerbations. Note that in this mediation analysis framework, the total effect (c) in part A is the sum of the direct (c’) and indirect paths (a→b) in part B, i.e., c = c’ + (a x b). Thus, because the sign of path a is negative while both b and c’ are positive, the total effect c (in panel A) is necessarily weaker in magnitude.

Subsequently, we tested whether rs140324259 was related to clinically-relevant COPD phenotypes, namely CB and AE. In the subset of SPIROMICS participants with complete phenotype, genotype, sputum MUC5B, and clinical outcomes data (n = 141–143), we found that rs140324259 was not associated with CB at baseline/enrollment (p = 0.25, S5 Table). rs140324259 was not associated with AE in the year prior to enrollment (p = 0.14, Fig 4A) unless we accounted for sputum MUC5B concentration (p = 0.02, Fig 4B and S6 Table). Surprisingly, in this analysis, we found that while MUC5B concentration was positively associated with AE (β_1_ = 0.45, p = 0.01, Fig 4B), the effect of rs140324259 genotype (β_2_ = 0.74, p = 0.02) was directionally opposite our expectations based on pQTL analysis. That is, the C allele of rs140324259, which was associated with lower MUC5B (γ = -0.77, p = 2.6 x10^-6^) and therefore would be expected to confer decreased risk of AE, was associated with increased risk of AE compared to the T allele.

These results suggested the possibility that rs140324259 may exert effects on AE through both direct and indirect paths, the latter via MUC5B (Fig 4B). To examine this further, we employed a mediation analysis approach, based on the framework developed by Baron and Kenny [22], in which the effect of rs140324259 on AE is modeled as the sum of direct (rs140324259 → AE) and indirect paths (rs140324259 → AE via MUC5B). We leveraged an intersection union test [23] to jointly test that both components of the indirect path (rs140324259 → MUC5B and MUC5B → AE) are statistically significant. Indeed, we found evidence that this is the case (p = 0.02), which is consistent with a model of partial mediation by MUC5B. Thus, overall, we conclude from these results that rs140324259 likely affects AE in two ways, both directly and indirectly, but with contrasting allele effects in each case, and overall the net effect is that rs140324259 C allele confers increased risk of AE. We note also that contrasting direction of effects of rs140324259 → MUC5B (-) and MUC5B → AE (+) likely explains why the magnitude of the association between rs140324259 and AE (not accounting for MUC5B) is weak and therefore not statistically significant (Fig 4A) [22].

We then examined associations between rs140324259 and clinical phenotypes in the larger SPIROMICS population for which genotype and clinical data exist but there is not sputum mucin concentration data (n≈1,250). In this sample, rs140324259 was associated with CB at baseline (p = 0.02, Table 2). Similar to results in the smaller subset of subjects described above, the C allele was associated with increased risk of CB (odds ratio (OR) = 1.42; 95% confidence interval (CI): 1.10–1.80). The effect of rs140324259 on AE in the larger SPIROMICS sample with clinical data was examined using both retrospectively and prospectively ascertained data. rs140324259 was not significantly associated with AE in the year prior to enrollment (S7 Table), nor in the year following enrollment (S8 Table). However, in both of these analyses, the results were suggestive and the direction of effect was again positive for the C allele.

**Table 2:** Logistic Regression Model of Chronic Bronchitis at Baseline and rs140324259 genotype (n = 1257).

Parameter	Odds Ratio	95% Confidence Interval
Age	0.99	0.98–1.00
Sex (M vs. F)	1.40	1.09–1.80
PC1	1.01	0.87–1.20
PC2	1.10	0.95–1.30
Smoking pack years	1.00	1.00–1.00
Current smoker	5.55	4.17–7.40
FEV1, % predicted	0.99	0.99–1.00
**rs140324259 (C vs. T)**	1.42	1.10–1.80

We then asked whether rs140324259 genotype was associated with AE over a period of three years of follow up. SPIROMICS participants’ exacerbation frequency was categorized based on a previous study as never (n = 433), inconsistent (n = 331) or consistent (n = 58) over the three years [24] (see Methods). Using a proportional odds model, we analyzed whether rs140324259 genotype distinguished never exacerbators versus inconsistent and consistent AE, and whether rs140324259 genotype distinguished consistent exacerbators versus never and inconsistent exacerbators. We found that rs140324259 genotype was associated with the former contrast (p = 0.03), with the C allele conferring increased risk of being either an inconsistent or consistent exacerbator (S9 and S10 Tables). rs140324259 genotype was not associated with the contrast between consistent exacerbators versus never and inconsistent exacerbators. To simplify the interpretation of the effect of rs140324259 on prospectively ascertained AE, we then dichotomized subjects into two groups, those who experienced AE (inconsistent and consistent) versus those who did not. As shown in Table 3, the rs140324259 C allele conferred increased risk of AE over three years of follow up (OR = 1.41; 95% CI: 1.02–1.94).

**Table 3:** Logistic Regression Model Comparing Exacerbators Versus Non-Exacerbators Based on Prospectively Ascertained Exacerbation Count Over a Three-Year Period (n = 822).

Parameter	Odds Ratio	95% Confidence Interval
Age	1.00	0.97–1.20
Sex (M vs F)	0.65	0.47–0.89
PC1	0.95	0.78–1.15
PC2	1.11	0.90–1.37
Smoking, pack years	1.01	1.00–1.01
Current smoker	1.22	0.86–1.74
Exacerbations, year prior to enrollment	1.96	1.54–2.49
FEV1, % predicted	0.97	0.96–0.98
**rs140324259 (C vs. T)**	1.41	1.02–1.94

### Replication analyses

Finally, we analyzed data from the COPDGene study population and UK Biobank in an attempt to replicate results from SPIROMICS. For COPDGene, we utilized phenotype data from COPD cases of European ancestry, and genotypes from whole genome sequencing. Sample sizes in these analyses ranged from 5300–5700 depending on the outcome. In this population of COPD cases, rs140324259 was not associated with CB (OR = 1.08 (95% CI: 0.94–1.24), nor AE (S11–S13 Tables), though we were unable to directly evaluate whether rs140324259 was associated with the exacerbation frequency categories (never, inconsistent, consistent) described in SPIROMICS. In the UK Biobank, however, we found that rs140324259 was associated with two CB-related phenotypes, namely bringing up phlegm/sputum/mucus on most days and cough on most days (Table 4). Importantly, the C allele was enriched in cases vs. controls for these two phenotypes, thus these results are directionally consistent with results from SPIROMICS. Results for other variants in LD with rs140324259 are shown in S14 Table. As UK Biobank results were not adjusted for smoking, we additionally assessed whether rs140324259 was associated with smoking. rs140324259 was either not associated with smoking history variables or was weakly associated, but in these cases the C allele frequency was higher in controls than cases (Table 4), suggesting that the associations between rs140324259 and CB-related phenotypes in the UK Biobank are unlikely to be mediated by, or confounded with, smoking.

**Table 4:** Association analysis results for lead MUC5B pQTL variant with CB-related phenotypes and smoking history in the UK Biobank.*

UK Biobank Phenotype	# cases/controls	rs140324259 C Allele Frequency Cases	rs140324259 C Allele Frequency Controls	p-value
Bring up phlegm/sputum/mucus on most days	9,250 / 97,072	0.150	0.143	9.80E-03
Cough on most days	14,606 / 91,635	0.149	0.143	4.50E-03
Current smoking status	43,192 / 375,625	0.141	0.143	9.40E-02
Ever smoked	253,507 / 165,353	0.143	0.143	9.73E-01
Smokes tobacco on all or most days	2,447 / 103,281	0.137	0.144	1.93E-01

*analysis limited to subjects of European Ancestry.

In contrast to the associations we detected between COPD phenotypes and the lead MUC5B pQTL variant, we did not detect any associations with the other MUC5B pQTL (rs10001928), nor the two MUC5AC pQTL (rs75401036 and rs16866419), in SPIROMICS, COPDGene, or the UK Biobank, as shown in S15–S17 Tables.

## Discussion

Using quantitative measurements of sputum mucin concentrations, we identified three genome-wide significant loci and one highly suggestive locus associated with MUC5AC or MUC5B. The strongest signal we detected, with rs140324259, accounted for a large percent of variation in MUC5B, and is independent of the common *MUC5B* promoter variant associated with IPF. Surprisingly, rs140324259 does not appear to be an eQTL for *MUC5B*, though we note that our sample size for eQTL analysis was not large and that the tagSNP we used is not in very high LD with rs140324259. One nearby variant, rs11604917, is intriguing given that it potentially disrupts binding of the transcription factor RBP-J, a key player in the Notch signaling pathway that determines ciliated vs. secretory cell fate in murine airways [19]. This could suggest that the MUC5B pQTL is a function of cell type composition of the airway epithelium, an idea supported by the lack of an association with gene expression. However, this variant is in low LD with rs140324259, and the association of rs11604917 with CB-related phenotypes in the UK Biobank was not nearly as strong as for rs140324259, arguing against a causal role for rs11604917. Additionally, rs11604917 genotype was not associated estimated secretory cell frequency in airway brush samples, though power was also limited in this analysis. Thus, in total, the mechanism underlying the MUC5B pQTL, including the causal variant(s), remains to be determined.

Given that previous studies have identified eQTL for *MUC5AC* [10–12] in asthma and *MUC5B* [13] in IPF located near the genes themselves (“local eQTL”), one potential *a priori* prediction could have been that these same variants would be associated with MUC5AC and MUC5B protein concentrations. This was not the case, even in the context of a regional association analysis (i.e., not a genome-wide significance threshold). This is perhaps not surprising for at least two reasons. First, there are clear differences between our study and the previous studies as a function of disease state (COPD vs. asthma vs. IPF) and anatomical location (upper vs. lower airways). Second, mucin protein concentration is the product of several pathways beyond just mucin gene transcription, including protein synthesis, post-translation modifications, packaging into vesicles, secretion, airway hydration via ion transport, and mucociliary clearance. Thus, one could reasonably expect that genetic variants that regulate any of these processes could be associated with mucin concentration. It remains to be determined whether any of the distal/off-chromosome pQTL identified here play a role in one or more of these pathways. That we did not identify any associations in/near genes with known roles in these processes suggests that either we were underpowered to detect these associations and/or that there is limited functional genetic variation in/near these genes.

Our analysis of rs140324259 genotype and clinical outcomes produced intriguing results in the SPIROMICS cohort, namely associations with CB and prospectively ascertained AEs. These results did not replicate in COPDGene, but we did find an association with sputum production and cough in a much larger dataset, the UK Biobank. These data argue in support of a role for the MUC5B pQTL in CB-related phenotypes. However, we acknowledge that the results of CB and AE association analyses with rs140324259 in SPIROMICS would not a survive multiple testing correction based on the number of outcomes/models we evaluated; in addition, we were unable to replicate these results in COPDGene, thus raising the potential that the results in SPIROMICS represent false positives. We note here that failure to replicate genetic associations with AE is unfortunately common [25], and future studies in which standardized definitions of AE can be employed will certainly facilitate the best comparisons across studies [25]. It is also worth noting that a previous study identified significant blood biomarkers of susceptibility for AE in SPIROMICS and separately in COPDGene, but there was essentially no overlap in associations between the two populations [26], which points to the difficulty in identifying reproducible predictors of exacerbations. The UK Biobank analysis, while supportive of our results, did not have the same degree of detailed respiratory phenotypes and was performed in a general population sample. Additional analyses adjusting for disease state and other covariates could be beneficial [27]. Further attempts to replicate these finding in other populations would also be useful, in particular to address the question of generalizability across populations of different genetic ancestries.

In aggregate, the results of association tests between rs140324259 and COPD phenotypes suggest an apparent paradox. While MUC5B concentration was positively associated with AE and CB in SPIROMICS, and the C allele was associated with significantly reduced MUC5B, the C allele overall was associated increased risk of AE and CB. This result suggests that higher expression of MUC5B may in fact be protective against AE and CB, perhaps by virtue of normalizing the ratio of elevated MUC5AC to MUC5B, making it more clearable, as has been suggested before in relationship to the IPF-associated variant rs35705950 [28].

A recent study also based in SPIROMICS showed that MUC5AC concentration is a stronger predictor of COPD initiation, disease progression, and exacerbations than MUC5B concentration [9]. As such, it is somewhat surprising that the MUC5AC pQTL we detected (rs75401036 and rs16866419) were not associated with the COPD phenotypes assessed in this study. In two cases (rs75401036), the minor allele frequency was quite low (2%), thus power to detect an association was lower as well. In contrast, rs16866419 had an allele frequency (10%) only slightly lower the lead MUC5B pQTL (11%), but was not as strongly associated with MUC5AC protein as rs140324259 was with MUC5B.

While we examined associations between loci associated with mucins, CB, and AE in COPD patients specifically, others have examined the genetics of CB/chronic mucin hypersecretion in combined analysis of the general population and patients with COPD [27,29] or in smokers without COPD [30]. In the study with COPD cases and the general population [29], the most consistent association signal was for rs6577641, which was also shown to act as an eQTL for the gene *SATB1*. In look up analysis, this variant was not associated with either sputum MUC5B concentration or CB in the SPIROMICS population, nor was the lead variant (rs10461985) from another study [30]. The most recent study reported an association of variants on proximal Chr 11 (near *MUC2*) with chronic sputum production using the same UK Biobank phenotype codes we used [27], but the LD between lead variant in that study (rs779167905) and rs140324259 is minimal (R^2^ = 0.08), making it unlikely that these are the same signals.

In summary, we identified pQTL for MUC5AC and MUC5B in sputum, demonstrating that common genetic variants influence these biomarkers. The lead MUC5B pQTL, rs140324259, was associated with CB and prospectively ascertained AE in SPIROMICS and was also associated with CB-related phenotypes in the UK Biobank. Additional studies are needed to determine how this variant influences MUC5B concentration in sputum and to further evaluate whether rs140324259 may be a biomarker of CB and AE susceptibility in COPD in other populations.

## Materials and methods

### Ethics statement

Subjects provided informed written consent to participate in the studies described here. Details and institutional review boards for each clinical site are provided in S2 File.

### Study subjects and genotype data

The primary analyses presented here are based on study participants in SPIROMICS (ClinicalTrials.gov Identifier: NCT01969344), and a schematic of the SPIROMICS datasets used here is shown in S1 Fig. The study design has been described previously [31]. SPIROMICS participants were genotyped using the Illumina OmniExpress Human Exome Beadchip [32]. Quality controls included testing for sex concordance and removal of SNPs with high genotype missing rates (>5%) and/or violations of Hardy Weinberg equilibrium at p < 1x10^-6^. Genotype imputation was performed using the Michigan Imputation Server [33] using haplotypes from Phase3 of the 1000 Genomes Project [34]. Study participants were categorized into either European ancestry (EA, N = 576 or African ancestry (AA, N = 132) groups based on principal components analysis of genotype data and comparisons to 1000Genomes data. We used an adaptive R^2^ threshold to filter imputed variants in each ancestry group based on the minor allele frequency (MAF). For each MAF interval, the R^2^ value was chosen such that the average R^2^ for variants with values larger than the threshold was at least 0.8 (S18 and S19 Tables). We limited our analyses to SNPs with minor allele counts >8, resulting in ∼10 million SNPs in EA and 12 million variants in AA subjects for association with total and specific mucin concentrations.

In COPDGene [35] (ClinicalTrials.gov Identifier: NCT00608764), genotype data for rs140324259 was obtained from whole genome sequencing performed through the TOPMed consortium [36]. Results from the UK Biobank data were obtained from the Pan-UK Biobank analysis (see further description below) [37].

### Sputum mucin phenotype data

Sputum mucin concentration: sputum induction and measurement methods have been previously reported [8,9,38]. In brief, hypertonic saline was used to induce sputum, which was then placed in a buffer containing 6 molar guanidine, and stored at 4 degrees. Total sputum mucin concentration was determined using a size exclusion chromatography / differential refractometry measurement approach. For a subset of subjects, MUC5AC and MUC5B concentration was determined using stable isotope labeled mass spectrometry [38]. Data were generated in two batches. In addition to SPIROMICS participants with COPD (n = 439), two additional sets of subjects were also included in the mucin analyses: non-smoking controls (n = 50), and smokers without COPD that are referred to as the “at-risk” group (n = 219). These subjects were included in genetic analysis of sputum mucin concentration but were not included in the analysis of COPD outcomes.

### Clinical/phenotype data

We analyzed data on two COPD phenotypes, namely CB and AE, in SPIROMICS. The CB phenotype was ascertained at the first study visit (“baseline”) and was categorized based on participants’ responses to questions regarding frequency of cough and mucus/phlegm production in the St. George’s Respiratory Questionnaire, as described in a previous publication [39]. More specifically, participants were categorized as having CB if they indicated they cough either most days a week or several days a week and bring up phlegm/sputum either most days a week or several days a week. If participants said they only cough or bring up phlegm with respiratory infection or not at all, they were categorized as negative for CB.

The analysis of AE was based on previous work from SPIROMICS [24,31] in which AE were defined as events that required health care utilization (i.e., office visit, hospital admission, or emergency department visit for a respiratory flare-up) involving the use of antibiotics and/or systemic corticosteroids. In COPDGene, the CB phenotype was based on chronic cough and phlegm production for ≥ 3 months/year for 2 consecutive years [5]. For AE, self-reported moderate-to-severe exacerbations in the year prior to enrollment and the number of moderate-to-severe exacerbations ascertained prospectively from longitudinal follow up data were examined. In the Pan-UK Biobank (https://pan.ukbb.broadinstitute.org/), we evaluated results of association analyses for two CB-related phenotypes, namely bringing up phlegm/sputum/mucus daily (yes vs. no, phenocode 22504) and coughing on most days (yes vs. no, phenocode 22502), as well as smoking history variables, which were assessed by questionnaire.

## Statistical models

### Sputum mucin concentration

Data on total and specific (MUC5AC and MUC5B) mucin concentrations were log-transformed prior to analysis. GWAS analysis was performed using version 0.5.0 of the ProbABEL software [40]. Analysis of total mucin concentration was conducted in each ancestry group separately (N = 576 EA and 132 AA), followed by a pooled analysis of both ancestry groups. In ancestry-specific analyses, main effect SNP models of each mucin phenotype included covariates for the top two principal components of ancestry (PC) obtained from EIGENSTRAT [41], age, sex, batch of mucin quantitation analysis, current smoking status, smoking pack-years, and CB. Results were not materially different when we included up to 10 genotype PCs. For MUC5AC and MUC5B, GWAS was performed in EA subjects only (N = 215) with the same covariates used for total mucin concentration. SNP-based heritability for MUC5AC and MUC5B was estimated on an LD-pruned set of markers from the genotyped data (subsetting to individuals with the relevant phenotype data) using GCTA version 1.92.1. We performed exploratory genome-wide interaction studies of SNP × smoking interactions in which we tested for the joint effects of SNP and SNP × smoking interactions (2 d.f. test) on mucin concentrations in models including the same covariates as above.

### eQTL analysis

Airway epithelial gene expression from 144 SPIROMICS participants was analyzed to test whether rs140324259 is an eQTL for *MUC5B* by performing a genome-wide eQTL mapping as described before in Kasela et al. [15]. Briefly, RNA-seq data from the airway epithelium was normalized, filtered, and transformed using inverse normal transformation. Genotype data was obtained from TOPMed (Freeze 9) [36]. The eQTL regression model for a given gene included sex, four genotype PCs, and 15 PEER factors (probabilistic estimation of expression residuals [42]) as covariates. eQTL mapping was performed using tensorQTL [43] and 10,000 permutations were used to control for multiple testing at false discovery rate (FDR) < 0.05. To look up the eQTL association with *MUC5B*, we used the proxy SNP rs55680540 because rs140324259 did not pass variant filter quality control.

### Cell type deconvolution

Using the same original dataset as was used for eQTL detection in bronchial epithelial brush samples, we performed cell type deconvolution using the non-negative least squares (NNLS) method (R package nnls) [44], which has been shown to perform well in comparison to other deconvolution methods [20]. Application of this method in a previous study of COPD subjects [21] showed good correlations between goblet cell proportions estimated in bulk sequencing data using NNLS and goblet cell numbers based on histological scoring. NNLS bulk deconvolution was carried out using cell type specific signatures derived from bronchial biopsies in six subjects with asthma (in which a larger proportion of goblet cells is identified than in healthy controls [45]). Cell type signature derivation of 14 known cell types was done in the scRNAseq data using AutoGeneS (Automatic gene selection using multi-objective optimization for RNA-seq deconvolution [46]). NNLS was then used to estimate the proportions of these cell types in the bronchial epithelial brush bulk RNA-seq samples of 137 SPIROMICS participants. Cell types included six epithelial cell populations (Ciliated cells, Goblet/Club cells, Submucosal Secretory cells, Basal cells, Basal cycling cells, and Ionocytes), two stromal cell populations (Endothelial cells and Fibroblasts), and six white blood cell populations (Mast cells, Dendritic cells, Alveolar macrophages, T cells, Monocytes, B cells).

We then performed analyses with both rs140324259, the lead MUC5B pQTL, and rs11604917, the variant in LD that is predicted to alter RBP-J transcription factor finding, testing for genotype associations with the frequency of goblet cells, ciliated cells, and basal cells, which are the cell types one would hypothesize should be most relevant to mucin gene expression (we did not test for association with submucosal secretory cells because all values were 0). We used a beta regression model with a data transformation applied to cell type frequencies (y’ = (y(n-1)+0.5)/n) to avoid having values of 0 or 1.

### Clinical phenotypes

Chronic Bronchitis (CB): Following the analyses of sputum mucin concentration data, we tested for an association between the lead variant for sputum MUC5B concentration (rs140324259) and CB in the larger SPIROMICS population (N = 1257). Logistic regression models were used for CB, accounting for the top two genotype PCs, age, sex, current smoking status, pack-years of smoking, and FEV1 (% predicted).

Acute Exacerbations (AE): Exacerbation outcomes were modeled using negative binomial regression models including the same covariates as above. Additionally, in the analysis of prospectively ascertained AE, we included AE in the year prior to enrollment as a predictor. Because prior work showed that exacerbation frequency among subjects with COPD in SPIROMICS is not stable [24], we leveraged a previously developed classification system which categorized SPIROMICS participants as never, inconsistent or consistent exacerbators using three years of follow up data [24]. Consistent exacerbators were subjects who experienced at least one acute exacerbation in each of the three years; subjects who had an exacerbation during some but not all of the three years of follow up were defined as inconsistent exacerbators. We analyzed the association between rs140324259 genotype and these three exacerbation groups using a proportional odds model, comparing (1) never exacerbators versus inconsistent and consistent exacerbators, and (2) consistent exacerbators versus never and inconsistent exacerbators. Based on the results of these analyses, we collapsed the exacerbation groups into two categories: ever (combining inconsistent and consistent exacerbators) vs. never exacerbators, then modeled this outcome using logistic regression with covariates for the top two genotype PCs, age, sex, current smoking status, pack-years of smoking, FEV1 (% predicted), and the number of AE in the year prior to enrollment.

### Mediation analysis

In the subset of SPIROMICS subjects for which there is complete phenotype data on genotype, sputum MUC5B, and clinical outcomes (N = 141), we tested for evidence of that MUC5B mediates an association between rs140324259 and AE (i.e. rs140324259 → MUC5B → AE), invoking the overall mediation analysis framework of Baron and Kenny [22].

We evaluated a direct path from rs140324259 → AE (c), and an indirect path from rs140324259 → MUC5B (a) and MUC5B → AE (b), while also examining the path from rs140324259 → AE conditional on MUC5B (c’). All regression models included age, sex, two ancestry PCs, current smoking status, pack-years of smoking, and FEV1 (% predicted) as predictors. For (a), we used a linear model for MUC5B in which rs140324259 was coded linearly (0,1,2). For (b), (c), and (c’), we used negative binomial regression models of AE that included rs140324259 (c), MUC5B (b), or both (c’). To formally test for mediation, we leveraged the SNP mediation intersection-union test (SMUT) [23] which jointly tests for non-zero parameter estimates from models for (a) and (b), which is equivalent to testing that a x b is not equal to 0 in the Baron and Kenny framework.

### Disclosure

The content is solely the responsibility of the authors and does not necessarily represent the official views of the National Heart, Lung, and Blood Institute or the National Institutes of Health.

## Supporting information

S1 Fig SPIROMICS participant data utilized in this study. Clinical data used includes FEV1, chronic bronchitis, and acute exacerbations. In GWAS models, all subjects were used; in models of clinical outcomes, subjects without COPD were removed. AA: African Ancestry, EA: European Ancestry. Note that while the figure suggests nested subsets of SPIROMICS data used in these analyses, in reality, there are varying degrees of overlap between subjects in mucin datasets and clinical dataset.
(PDF)

S2 Fig Distributions of sputum mucin concentrations. Distributions for total mucin (A, n = 576 EA/132 AA), MUC5AC (B, n = 215 EA), and MUC5B (C, n = 215) as a function of smoking history (left) and GOLD stage (right).
(PDF)

S3 Fig GWAS results for sputum total mucin concentration in EA subjects (N = 576). A. Manhattan plot. N = 576. B. Corresponding quantile-quantile plot.
(PDF)

S4 Fig GWAS results for sputum total mucin concentration in AA subjects (N = 132). A. Manhattan plot. N = 132. B. Corresponding quantile-quantile plot.
(PDF)

S5 Fig GWAS results for sputum total mucin concentration in combined analysis of EA + AA subjects (N = 708). A. Manhattan plot. B. Corresponding quantile-quantile plot.
(PDF)

S6 Fig Quantile-quantile plots for MUC5AC (A) and MUC5B (B) GWAS results in EA subjects (n = 215). (PDF)

S7 Fig Locus zoom plots for mucin pQTL that act in trans. MUC5AC pQTL on chromosome 7 (A) and MUC5B pQTL on chromosome 4 (B). The suggestive locus for MUC5AC on Chromosome 2 is also shown (C).
(PDF)

S8 Fig eQTL analysis for MUC5B in 144 SPIROMICS participants. A. Airway brush RNA-seq data was analyzed in relation to a proxy SNP for rs140324259, rs55680540. MUC5B expression data is plotted as residuals from a model containing four genotype PCs, age, sex, and 15 PEER factors. B. Results of scan for other local eQTL for MUC5B, with rs55680540 highlighted. Blue horizontal line corresponds to regional multi-testing threshold.
(PDF)

S9 Fig Putative disruption of RBP-J binding to a region upstream of mucin gene cluster on Chr 11. A. Location of lead MUC5B pQTL variant, rs140324259, and rs11604917. Note that rs11604917 (T->C) lies in the first position of a putative RBP-J binding motif. B. Consensus motif for RBP-J. Note that the first position is essentially invariant. C. Location of rs11604917 relative to enhancer marks detected in the airway epithelia cell line A549.
(PDF)

S1 Table Sputum MUC5AC and MUC5B pQTL. (XLSX)

S2 Table Transcription factor binding sites predicted to be altered by MUC5B pQTL variants. (XLSX)

S3 Table Potential Epigenomic Effects of MUC5B pQTL variants from Haploreg. (XLSX)

S4 Table Association Testing for rs11604917 and cell type proportions estimated from airway brush Bulk-RNA seq data using non-negative least squares deconvolution. (XLSX)

S5 Table Logistic Regression Model of Chronic Bronchitis and rs140324259 among MUC5B Subset of SPIROMICS. (XLSX)

S6 Table Negative Binomial Regression Model of Exacerbation Count in Year Prior to Enrollment Among COPD Subjects with Sputum MUC5B Concentration Data. (XLSX)

S7 Table Negative Binomial Regression Model of Exacerbation Count in Year Prior to Enrollment. (XLSX)

S8 Table Negative Binomial Regression Model of Exacerbation Count in Year Following Enrollment. (XLSX)

S9 Table Proportional Odds Model of Exacerbation Categories and rs140324259 Genotype. (XLSX)

S10 Table Odds Ratios from Proportional Odds Model of Exacerbation Categories and rs140324259 Genotype. (XLSX)

S11 Table Logistic Regression Model of Chronic Bronchitis and rs140324259 in COPDGene. (XLSX)

S12 Table Negative Binomial Regression Model of Exacerbation Count in Year Prior to Enrollment in COPDGene. (XLSX)

S13 Table Negative Binomial Regression Model of Prospectively Ascertained Exacerbations in COPDGene. (XLSX)

S14 Table Association analysis results for other MUC5B pQTL SNPs with CB related phenotypes and smoking history in the UK Biobank. (XLSX)

S15 Table Regression model outout for other mucin pQTL and COPD Phenotypes in SPIROMICS. (XLSX)

S16 Table Regression model outout for other mucin pQTL and COPD Phenotypes in COPDGene. (XLSX)

S17 Table Association analysis results for other mucin pQTL SNPs with CB related phenotypes and smoking history in the UK Biobank. (XLSX)

S18 Table Imputation Quality Among Subjects of European Ancestry. (XLSX)

S19 Table Imputation Quality Among Subjects of African Ancestry. (XLSX)

S1 File List of Supporting Files. (DOCX)

S2 File Institutional Review Board Approval Documentation for SPIROMICS and COPDGene. (DOCX)

S3 File Data dictionary for S4 File. (XLSX)

S4 File Data used for results in Tables 1, 2, S7 and S8 and S2 Fig. (XLSX)

S5 File Data dictionary for S6 File. (XLSX)

S6 File Data used for analyses in Tables 3 and S9 and S10. (XLSX)

S7 File Data dictionary for S8 File. (XLSX)

S8 File Data used for analyses in Figs 2, 3, 4 and S8 and S5 and S6 Tables and S8 Fig. (XLSX)

S9 File Data dictionary for S10 File. (XLSX)

S10 File Data used for analyses in S4 Table. (XLSX)

S11 File R Code to reproduce the analyses from Fig 4, Tables 2 and S4–S8. (R)

S12 File SAS code to reproduce the analyses from Tables 3, S9 and S10. (SAS)
