# Supplementary material for: Genetic regulators of sputum mucin concentration and their associations with COPD phenotypes
Source: PLoS Genet. 2023 Jun 23;19(6):e1010445. doi: 10.1371/journal.pgen.1010445 (PMC10325042; doi:10.1371/journal.pgen.1010445)
Supplement: S1 Fig — Clinical data used includes FEV1, chronic bronchitis, and acute exacerbations. In GWAS models, all subjects were used; in models of clinical outcomes, subjects without COPD were removed. AA: African Ancestry, EA: European Ancestry. Note that while the figure suggests nested subsets of SPIROMICS data used in these analyses, in reality, there are varying degrees of overlap between subjects in mucin datasets and clinical dataset. (PDF) [file pgen.1010445.s001.pdf]

S1 Figure

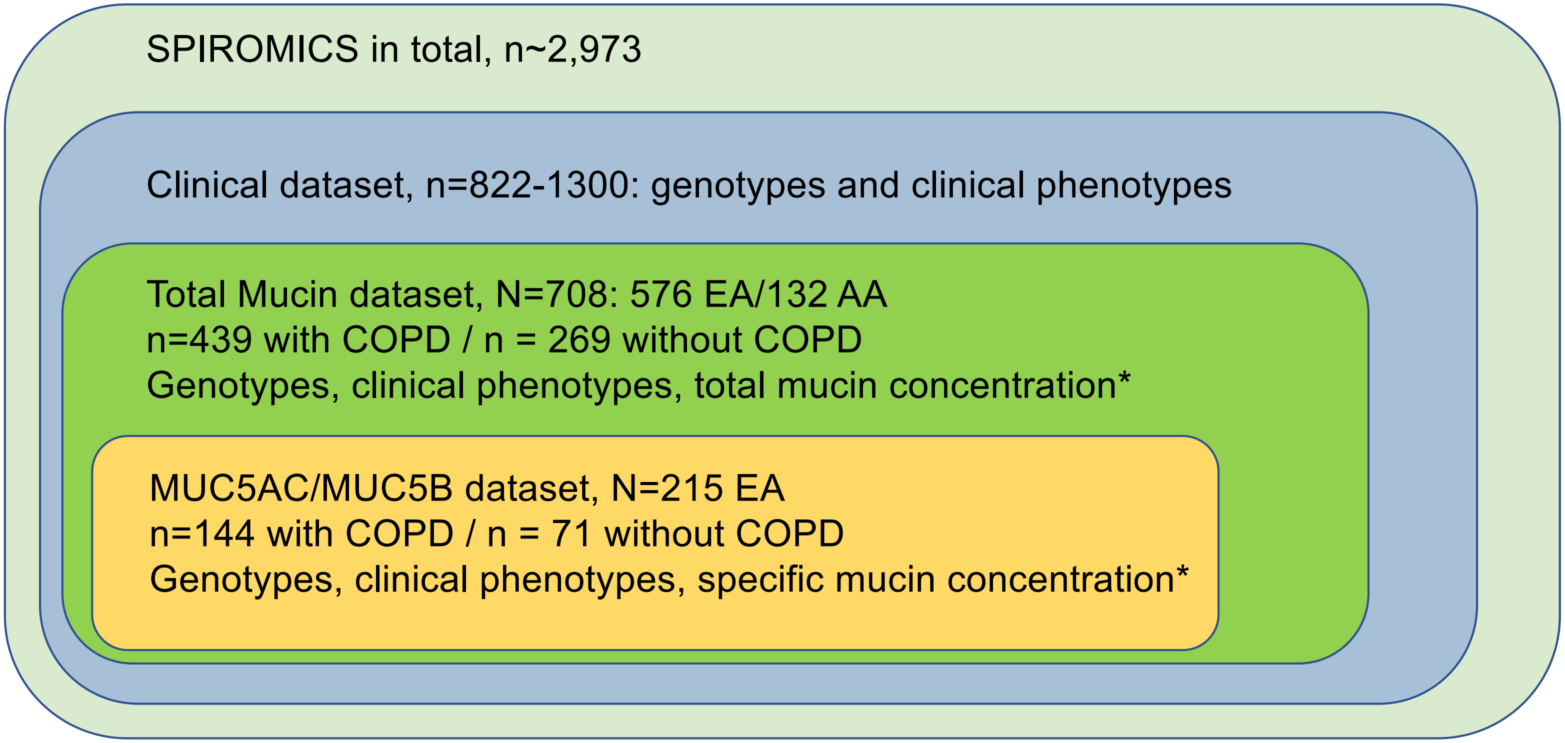

S1 Figure. SPIROMICS participant data utilized in this Study. Clinical data used includes FEV1, chronic bronchitis, and acute exacerbations. In GWAS models, all subjects were used; in models of clinical outcomes, subjects without COPD were removed. AA: African Ancestry, EA: European Ancestry. Note that while the figure suggests nested subsets of SPIROMICS data used in these analyses, in reality, there are varying degrees of overlap between subjects in mucin datasets and clinical dataset.
