# Supplementary material for: Genetic regulators of sputum mucin concentration and their associations with COPD phenotypes
Source: PLoS Genet. 2023 Jun 23;19(6):e1010445. doi: 10.1371/journal.pgen.1010445 (PMC10325042; doi:10.1371/journal.pgen.1010445)
Supplement: S2 Fig — Distributions for total mucin (A, n = 576 EA/132 AA), MUC5AC (B, n = 215 EA), and MUC5B (C, n = 215) as a function of smoking history (left) and GOLD stage (right). (PDF) [file pgen.1010445.s002.pdf]

S2 Figure

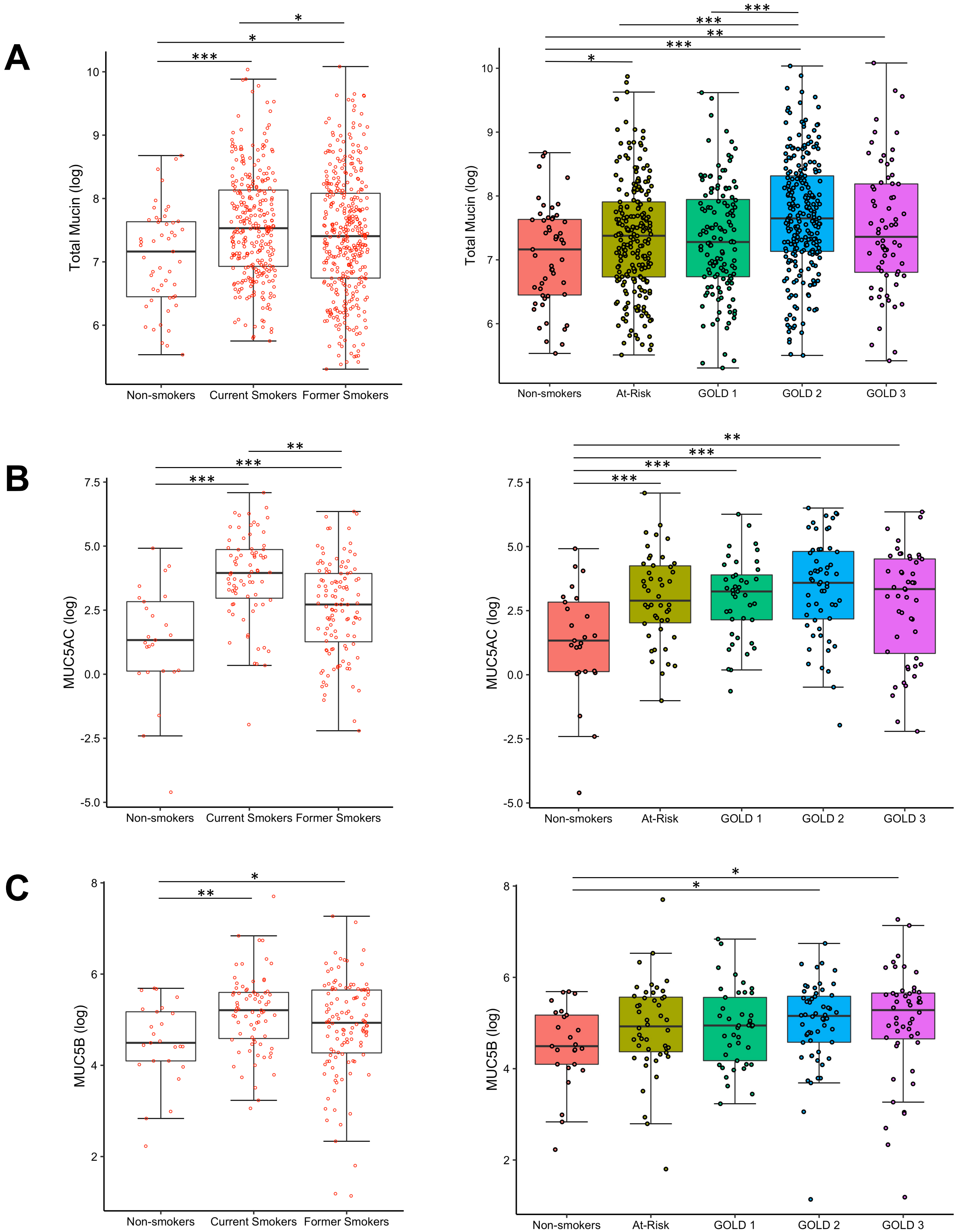

S2 Fig. Distributions of sputum mucin concentrations. Distributions for total mucin (A, n=576 EA/132 AA), MUC5AC (B, n=215 EA), and MUC5B (C, n=215) as a function of smoking history (left) and GOLD stage (right).
