# Supplementary material for: Genetic regulators of sputum mucin concentration and their associations with COPD phenotypes
Source: PLoS Genet. 2023 Jun 23;19(6):e1010445. doi: 10.1371/journal.pgen.1010445 (PMC10325042; doi:10.1371/journal.pgen.1010445)
Supplement: S3 Fig — A. Manhattan plot. N = 576. B. Corresponding quantile-quantile plot. (PDF) [file pgen.1010445.s003.pdf]

S3 Figure

A

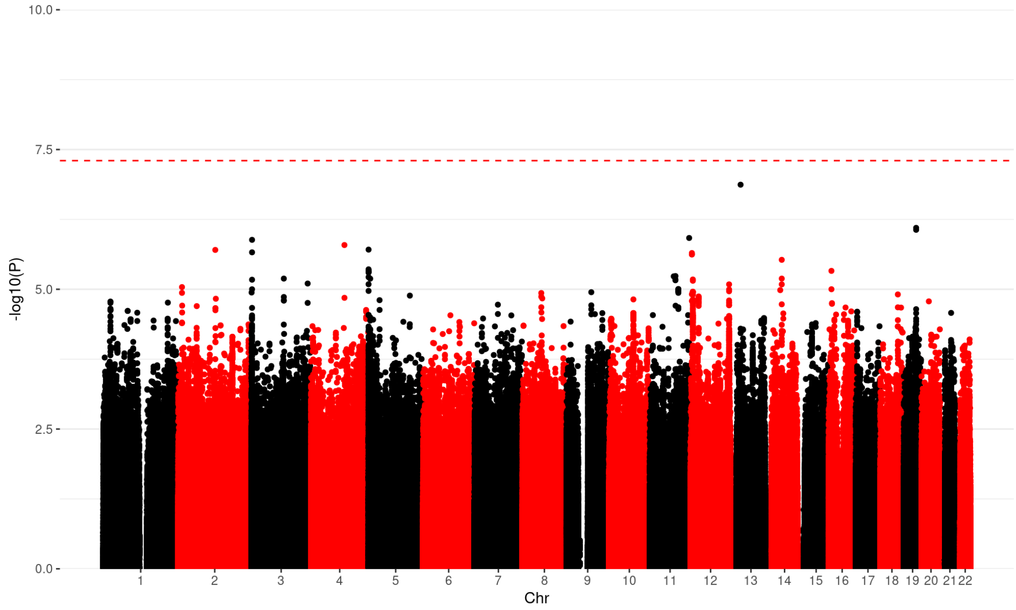

B

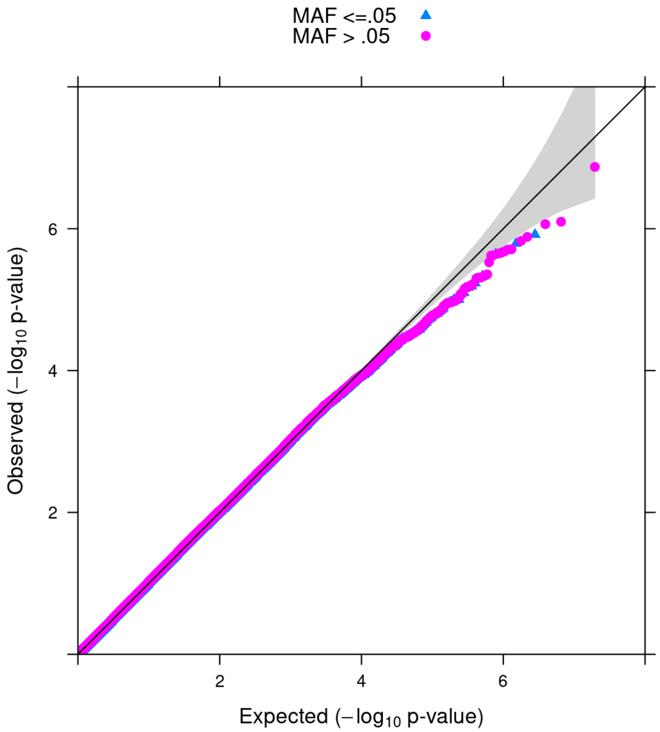

S3 Fig. GWAS results for sputum total mucin concentration in EA subjects (N=576). A. Manhattan plot. N=576.
B. Corresponding quantile-quantile plot.
