# Supplementary material for: Genetic regulators of sputum mucin concentration and their associations with COPD phenotypes
Source: PLoS Genet. 2023 Jun 23;19(6):e1010445. doi: 10.1371/journal.pgen.1010445 (PMC10325042; doi:10.1371/journal.pgen.1010445)
Supplement: S4 Fig — A. Manhattan plot. N = 132. B. Corresponding quantile-quantile plot. (PDF) [file pgen.1010445.s004.pdf]

S4 Figure

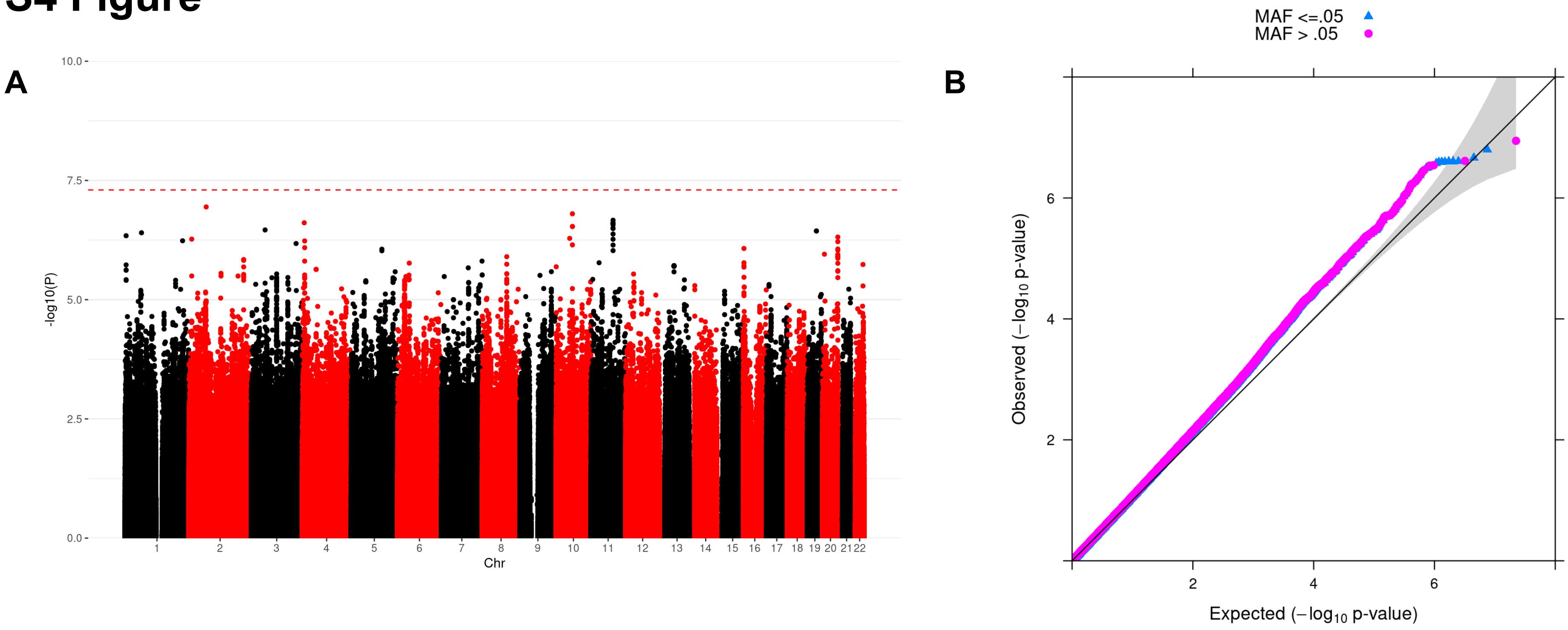

S4 Fig. GWAS results for sputum total mucin concentration in AA subjects (N=132). A. Manhattan plot. N=132.
B. Corresponding quantile-quantile plot.
