# Supplementary material for: Genetic regulators of sputum mucin concentration and their associations with COPD phenotypes
Source: PLoS Genet. 2023 Jun 23;19(6):e1010445. doi: 10.1371/journal.pgen.1010445 (PMC10325042; doi:10.1371/journal.pgen.1010445)
Supplement: S5 Fig — A. Manhattan plot. B. Corresponding quantile-quantile plot. (PDF) [file pgen.1010445.s005.pdf]

S5 Figure

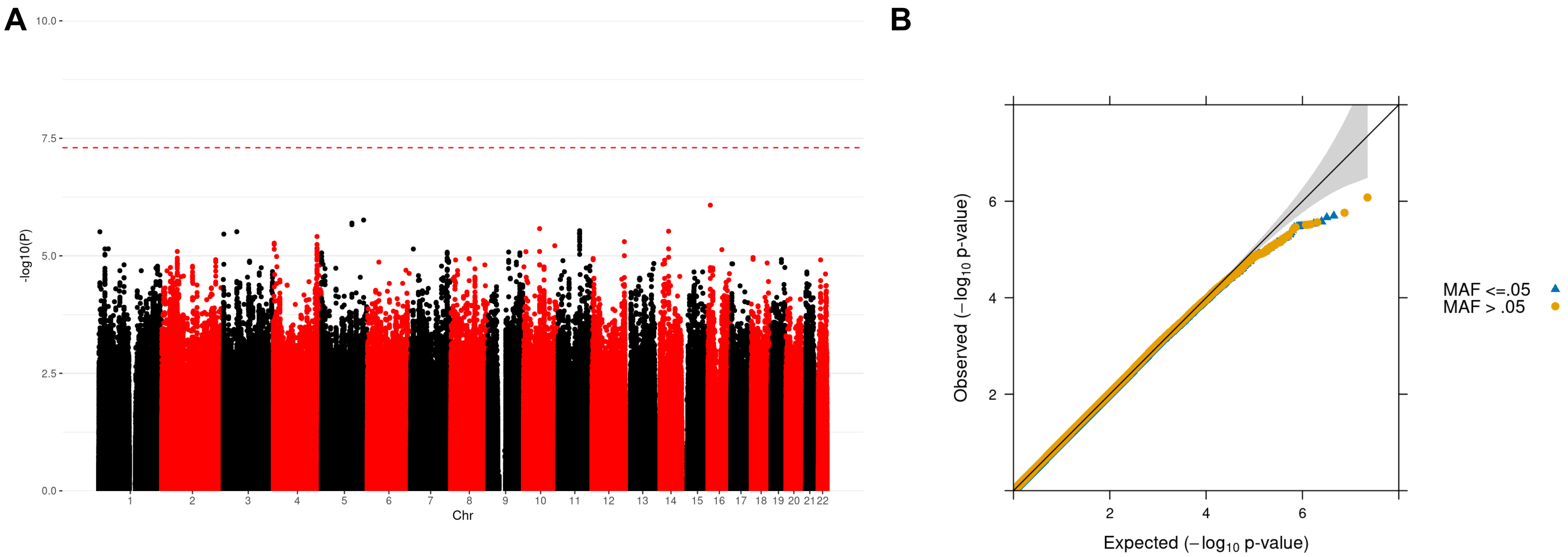

S5 Figure. GWAS results for sputum total mucin concentration in combined analysis of EA + AA subjects (N=708).
A. Manhattan plot. **B.** Corresponding quantile-quantile plot.
