# Supplementary figures and images for: Genetic regulators of sputum mucin concentration and their associations with COPD phenotypes
Source: PLoS Genet. 2023 Jun 23;19(6):e1010445. doi: 10.1371/journal.pgen.1010445 (PMC10325042; doi:10.1371/journal.pgen.1010445)

## S6 Figure

**A**

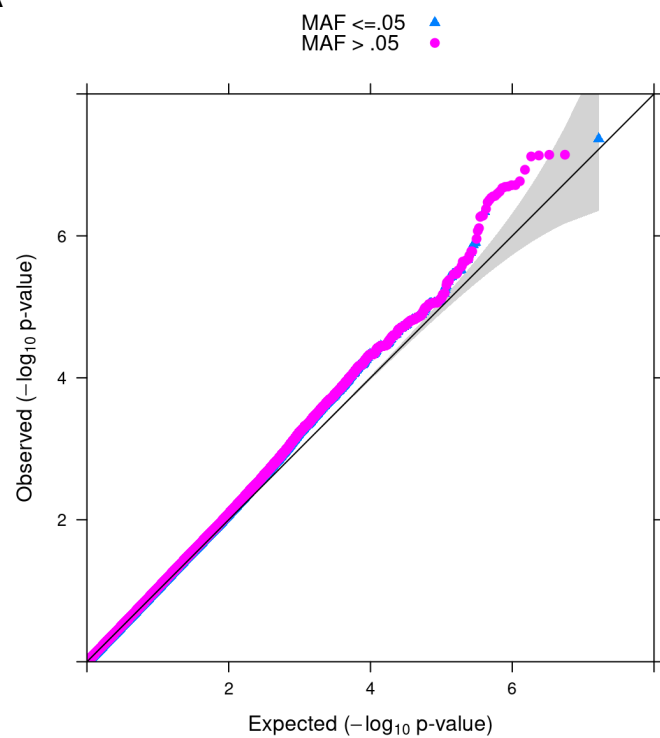

**B**

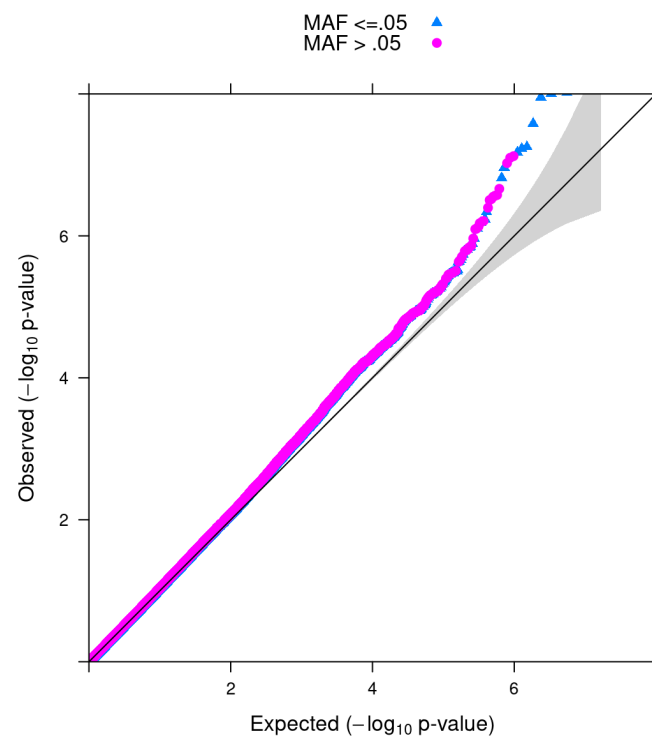

**S6 Fig. Quantile-quantile plots for MUC5AC (A) and MUC5B (B) GWAS results in EA subjects (n=215).**

Supplement: S6 Fig — (PDF) [file pgen.1010445.s006.pdf]
