# Supplementary material for: Genetic regulators of sputum mucin concentration and their associations with COPD phenotypes
Source: PLoS Genet. 2023 Jun 23;19(6):e1010445. doi: 10.1371/journal.pgen.1010445 (PMC10325042; doi:10.1371/journal.pgen.1010445)
Supplement: S7 Fig — MUC5AC pQTL on chromosome 7 (A) and MUC5B pQTL on chromosome 4 (B). The suggestive locus for MUC5AC on Chromosome 2 is also shown (C). (PDF) [file pgen.1010445.s007.pdf]

S7 Figure

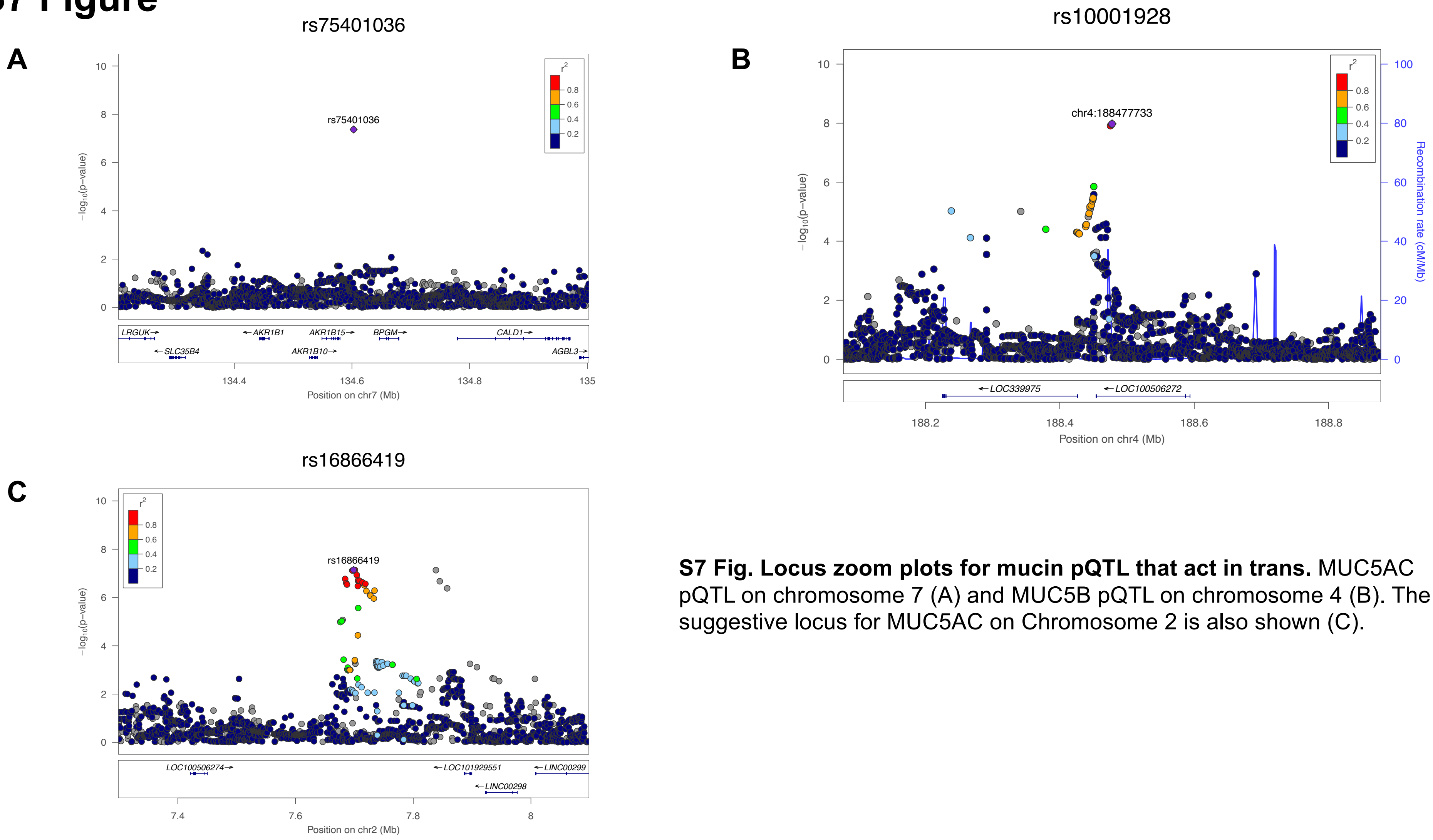

S7 Fig. Locus zoom plots for mucin pQTL that act in trans. MUC5AC pQTL on chromosome 7 (A) and MUC5B pQTL on chromosome 4 (B). The suggestive locus for MUC5AC on Chromosome 2 is also shown (C).
