# Supplementary material for: Genetic regulators of sputum mucin concentration and their associations with COPD phenotypes
Source: PLoS Genet. 2023 Jun 23;19(6):e1010445. doi: 10.1371/journal.pgen.1010445 (PMC10325042; doi:10.1371/journal.pgen.1010445)
Supplement: S8 Fig — A. Airway brush RNA-seq data was analyzed in relation to a proxy SNP for rs140324259, rs55680540. MUC5B expression data is plotted as residuals from a model containing four genotype PCs, age, sex, and 15 PEER factors. B. Results of scan for other local eQTL for MUC5B, with rs55680540 highlighted. Blue horizontal line corresponds to regional multi-testing threshold. (PDF) [file pgen.1010445.s008.pdf]

S8 Figure

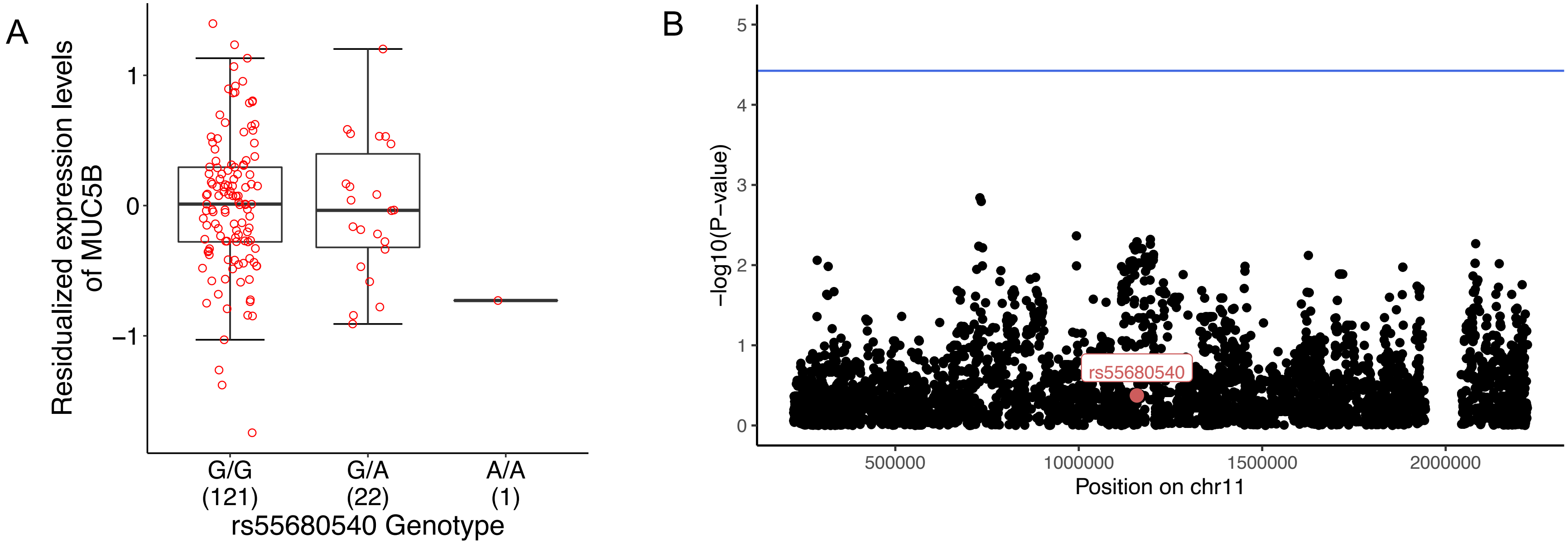

S8 Fig. eQTL analysis for *MUC5B* in 144 SPIROMICS participants. A. Airway brush RNA-seq data was analyzed in relation to a proxy SNP for rs140324259, rs55680540. MUC5B expression data is plotted as residuals from a model containing four genotype PCs, age, sex, and 15 PEER factors. **B.** Results of scan for other local eQTL for MUC5B, with rs55680540 highlighted. Blue horizontal line corresponds to regional multi-testing threshold.
