# Supplementary material for: Genetic regulators of sputum mucin concentration and their associations with COPD phenotypes
Source: PLoS Genet. 2023 Jun 23;19(6):e1010445. doi: 10.1371/journal.pgen.1010445 (PMC10325042; doi:10.1371/journal.pgen.1010445)
Supplement: S9 Fig — A. Location of lead MUC5B pQTL variant, rs140324259, and rs11604917. Note that rs11604917 (T->C) lies in the first position of a putative RBP-J binding motif. B. Consensus motif for RBP-J. Note that the first position is essentially invariant. C. Location of rs11604917 relative to enhancer marks detected in the airway epithelia cell line A549. (PDF) [file pgen.1010445.s009.pdf]

S9 Figure

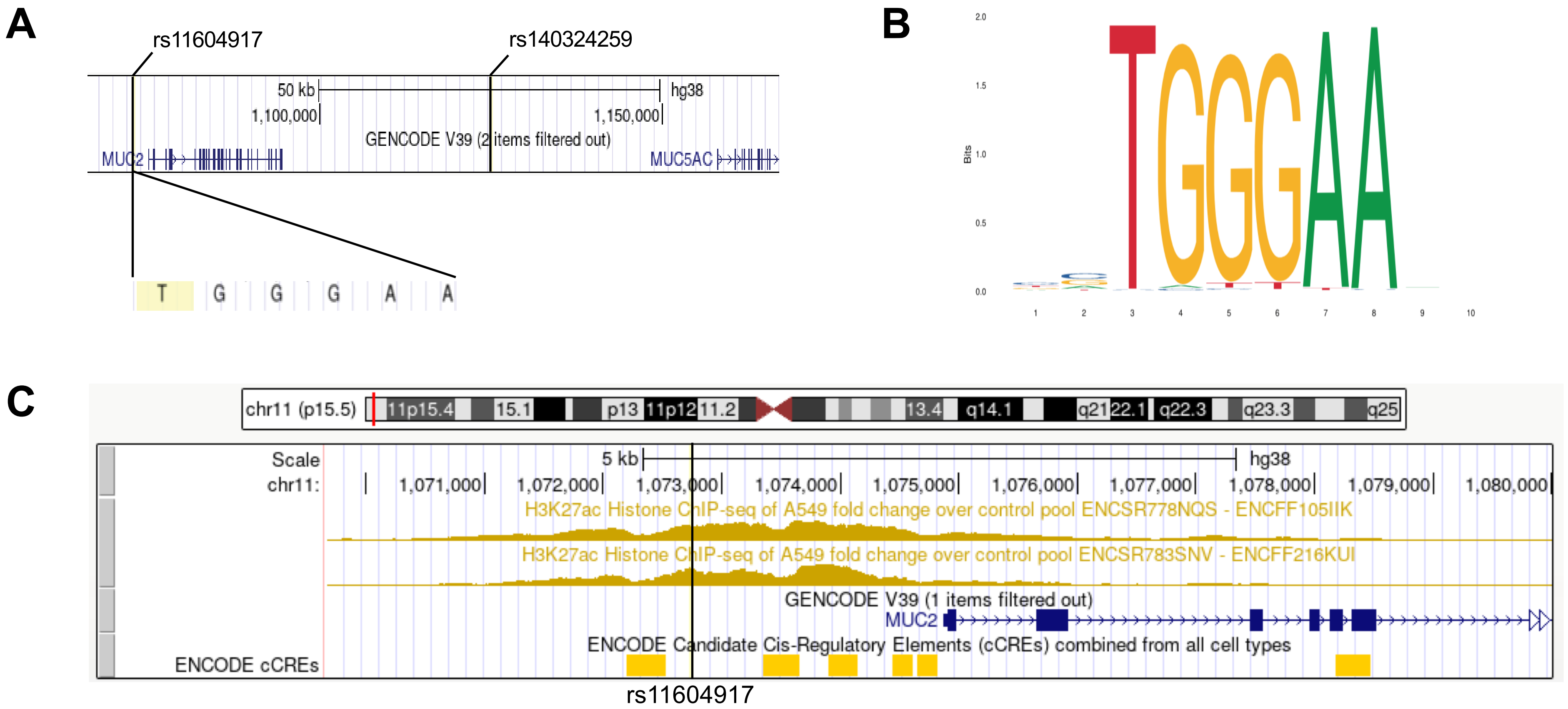

S9 Fig. Putative disruption of RBP-J binding to a region upstream of mucin gene cluster on Chr 11. A. Location of lead MUC5B pQTL variant, rs140324259, and rs11604917. Note that rs11604917 (T->C) lies in the first position of a putative RBP-J binding motif. **B.** Consensus motif for RBP-J. Note that the first position is essentially invariant. **C.** Location of rs11604917 relative to enhancer marks detected in the airway epithelia cell line A549.
