# Supplementary material for: Genetic regulators of sputum mucin concentration and their associations with COPD phenotypes
Source: PLoS Genet. 2023 Jun 23;19(6):e1010445. doi: 10.1371/journal.pgen.1010445 (PMC10325042; doi:10.1371/journal.pgen.1010445)
Supplement: S1 File — (DOCX) [file pgen.1010445.s029.docx]

**S1 File. List of Supporting Files**

| **Filename** | **Content** |
| --- | --- |
| S2_File.docx | Institutional Review Board Approval Documentation for SPIROMICS and COPDGene |
| S3_File.xlsx | Data dictionary for S4_File.xlsx |
| S4_File.xlsx | Data used for results in Tables 1 and 2, S7 and S8 Tables, and S2 Figure |
| S5_File.xlsx | Data dictionary for S6_File.xlsx |
| S6_File.xlsx | Data used for analyses in Table 3 and S9 and S10 Tables |
| S7_File.xlsx | Data dictionary for S8_file.xlsx |
| S8_File.xlsx | Data used for analyses in Figures 2, 3, and 4, S5 and S6 Tables, and S8 Fig |
| S9_File.xlsx | Data dictionary for S10_File.xlsx |
| S10_File.xlsx | Data used for analyses in S4_Table |
| S11_File.R | R Code to reproduce the analyses from Figure 4, Table 2, and S4-S8 Tables |
| S12_File.sas | SAS code to reproduce the analyses from Table 3 and S9 and S10 Tables |
