# Supplementary material for: Genetic regulators of sputum mucin concentration and their associations with COPD phenotypes
Source: PLoS Genet. 2023 Jun 23;19(6):e1010445. doi: 10.1371/journal.pgen.1010445 (PMC10325042; doi:10.1371/journal.pgen.1010445)
Supplement: S2 File — (DOCX) [file pgen.1010445.s030.docx]

**S2 File**

| **Institutional Review Board Approval Documentation for SPIROMICS and COPDGene** | | |
| --- | --- | --- |
| **Participating Center** | **Institution Title for Review Board** | **Protocol Number** |
| **SPIROMICS** |  |  |
| Columbia University Medical Center | Columbia University Medical Center IRB | IRB-AAAE9315 |
| Johns Hopkins University | Johns Hopkins Medicine Institutional Review Boards (JHM IRB) | NA_00035701 / CIR00004922 |
| National Jewish Health | National Jewish IRB | HS-2678 |
| Temple University | Temple University Office for Human Subjects Protections Institutional Review Board | 21416 |
| University of Alabama at Birmingham | The University of Alabama at Birmingham Institutional Review Board for Human Use | F120906004 |
| University of California, Los Angeles | UCLA Office of the Human Research Protection Program | 10-001740-CR-00004 |
| University of California, San Francisco | UCSF Human Research Protection Program, Committee on Human Research | 10-03169 |
| University of Illinois at Chicago | UIC Office for the Protection of Research Subjects (OPRS) | 2013-0939 |
| University of Iowa | The University of Iowa Human Subjects Office/Institutional Review Board (IRB) | 201308719 |
| University of Michigan | Medical School Institutional Review Board (IRBMED) | HUM00036346 |
| University of North Carolina at Chapel Hill | UNC-CH Office of Human Research Ethics (OHRE) Non-Biomedical IRB | 10-0048 |
| University of Utah | The University of Utah Institutional Review Board | 00027298 |
| Wake Forest University | Wake Forest University Health Sciences Office of Research Institutional Review Board | IRB00012805 |
|  |  |  |
| **COPDGene** |  |  |
| National Jewish Health | National Jewish IRB | HS-1883a |
| Brigham and Women’s Hospital | Partners Human Research Committee | 2007-P-000554/2; BWH |
| Baylor College of Medicine | Institutional Review Board for Baylor  College of Medicine and Affiliated Hospitals | H-22209 |
| Michael E. DeBakey VAMC | Institutional Review Board for Baylor College of Medicine and Affiliated Hospitals | H-22202 |
| Columbia University Medical Center | Columbia University Medical Center IRB | IRB-AAAC9324 |
| Duke University Medical Center | The Duke University Health System Institutional Review Board for Clinical Investigations (DUHS IRB) | Pro00004464 |
| Johns Hopkins University | Johns Hopkins Medicine Institutional Review Boards (JHM IRB) | NA_00011524 |
| Los Angeles Biomedical Research Institute | The John F. Wolf, MD Human Subjects Committee of Harbor-UCLA Medical Center | 12756-01 |
| Morehouse School of Medicine | Morehouse School of Medicine Institutional Review Board | 07-1029 |
| Temple University | Temple University Office for Human Subjects Protections Institutional Review Board | 11369 |
| University of Alabama at Birmingham | The University of Alabama at Birmingham Institutional Review Board for Human Use | FO70712014 |
| University of California, San Diego | University of California, San Diego Human Research Protections Program | 070876 |
| University of Iowa | The University of Iowa Human Subjects Office | 200710717 |
| Ann Arbor VA | VA Ann Arbor Healthcare System IRB | PCC 2008-110732 |
| University of Minnesota | University of Minnesota Research Subjects’ Protection Programs (RSPP) | 0801M24949 |
| University of Pittsburgh | University of Pittsburgh Institutional Review Board | PRO07120059 |
| University of Texas Health Sciences Center at San Antonio | UT Health Science Center San Antonio Institutional Review Board | HSC20070644H |
| Health Partners Research Foundation | Health Partners Research Foundation Institutional Review Board | 07-127 |
| University of Michigan | Medical School Institutional Review Board (IRBMED) | HUM00014973 |
| Minneapolis VA Medical Center | Minneapolis VAMC IRB | 4128-A |
| Reliant Clinic | Institutional Review Board/Research Review Committee Saint Vincent Hospital – Fallon Clinic – Fallon Community Health Plan | 1143 |
